# Effect of land use, habitat suitability, and hurricanes on the population connectivity of an endemic insular bat

**DOI:** 10.1038/s41598-021-88616-7

**Published:** 2021-04-27

**Authors:** Camilo A. Calderón-Acevedo, Armando Rodríguez-Durán, J. Angel Soto-Centeno

**Affiliations:** 1 Department of Biological Sciences, Rutgers University, Newark, USA; 2 Universidad Interamericana de Puerto Rico, Bayamón, USA; 3 Department of Earth and Environmental Sciences, Rutgers University, Newark, USA; 4 Department of Mammalogy, American Museum of Natural History, New York, USA

**Keywords:** Conservation biology, Ecological modelling

## Abstract

Urbanization and natural disasters can disrupt landscape connectivity, effectively isolating populations and increasing the risk of local extirpation particularly in island systems. To understand how fragmentation affects corridors among forested areas, we used circuit theory to model the landscape connectivity of the endemic bat *Stenoderma rufum* within Puerto Rico. Our models combined species occurrences, land use, habitat suitability, and vegetation cover data that were used either as resistance (land use) or conductance layers (habitat suitability and vegetation cover). Urbanization affected connectivity overall from east to west and underscored protected and rustic areas for the maintenance of forest corridors. Suitable habitat provided a reliable measure of connectivity among potential movement corridors that connected more isolated areas. We found that intense hurricanes that disrupt forest integrity can affect connectivity of suitable habitat. Some of the largest protected areas in the east of Puerto Rico are at an increasing risk of becoming disconnected from more continuous forest patches. Given the increasing rate of urbanization, this pattern could also apply to other vertebrates. Our findings show the importance of maintaining forest integrity, emphasizing the considerable conservation value of rustic areas for the preservation of local biodiversity.

## Introduction

Untangling the processes that affect biodiversity is a fundamental endeavor in ecology and conservation biology. Due to the fast pace of global change, processes like habitat conversion or fragmentation during the Anthropocene have become increasingly important as potential drivers of biodiversity loss in terrestrial ecosystems^1–3^. Island systems are in special need for conservation assessments because many islands are considered biodiversity hotspots^4–7^. Yet, a significant proportion of native insular biodiversity is often threatened by an ever-growing human footprint that has led to a significant loss of habitat^8–13^. Terrestrial vertebrates on islands in the Caribbean are further threatened by an increased frequency in extreme climatic events such as hurricanes, which can have short- and long-term impacts on their populations^14^. Therefore, evaluating the events that may lead to population changes in island systems is key to assessing how biological communities are structured across the landscape and examine their responses to anthropogenic and natural disturbances. Understanding how the remaining habitat connects across the landscape provides an exceptional opportunity to address issues of habitat conversion and fragmentation to examine how potential biological corridors may aid in conservation efforts. The Caribbean island of Puerto Rico, itself a small archipelago of some 140 small islands and cays, contains seven different types of forest habitats^15,16^. These forests are subject to various levels of anthropogenic and natural pressures across the island derived from urban development concentrated in the northeast and south-central areas^17^, and agricultural development located primarily in the west and southwest. Across Puerto Rico, there are 118 protected and specially protected rustic areas with an extension of over 3300 km^2^. Protected areas correspond to private or government land that is under conservation management, and are classified as commonwealth forests, nature reserves, federal reserves, and non-governmental protected areas^18–20^. On the other hand, rustic areas are forested areas that lack conservation management but due to their ecological, anthropological, cultural and agricultural value, are somewhat protected from development and urbanization, acting as habitat buffer zones^18,21,22^. Thus, rustic areas help connect protected areas by providing isolation from disturbance (i.e., urbanized areas or main roads), yet there is the potential for rustic areas to become agricultural valleys as the demand for natural resources grows^18,22^.

**Figure 1:**
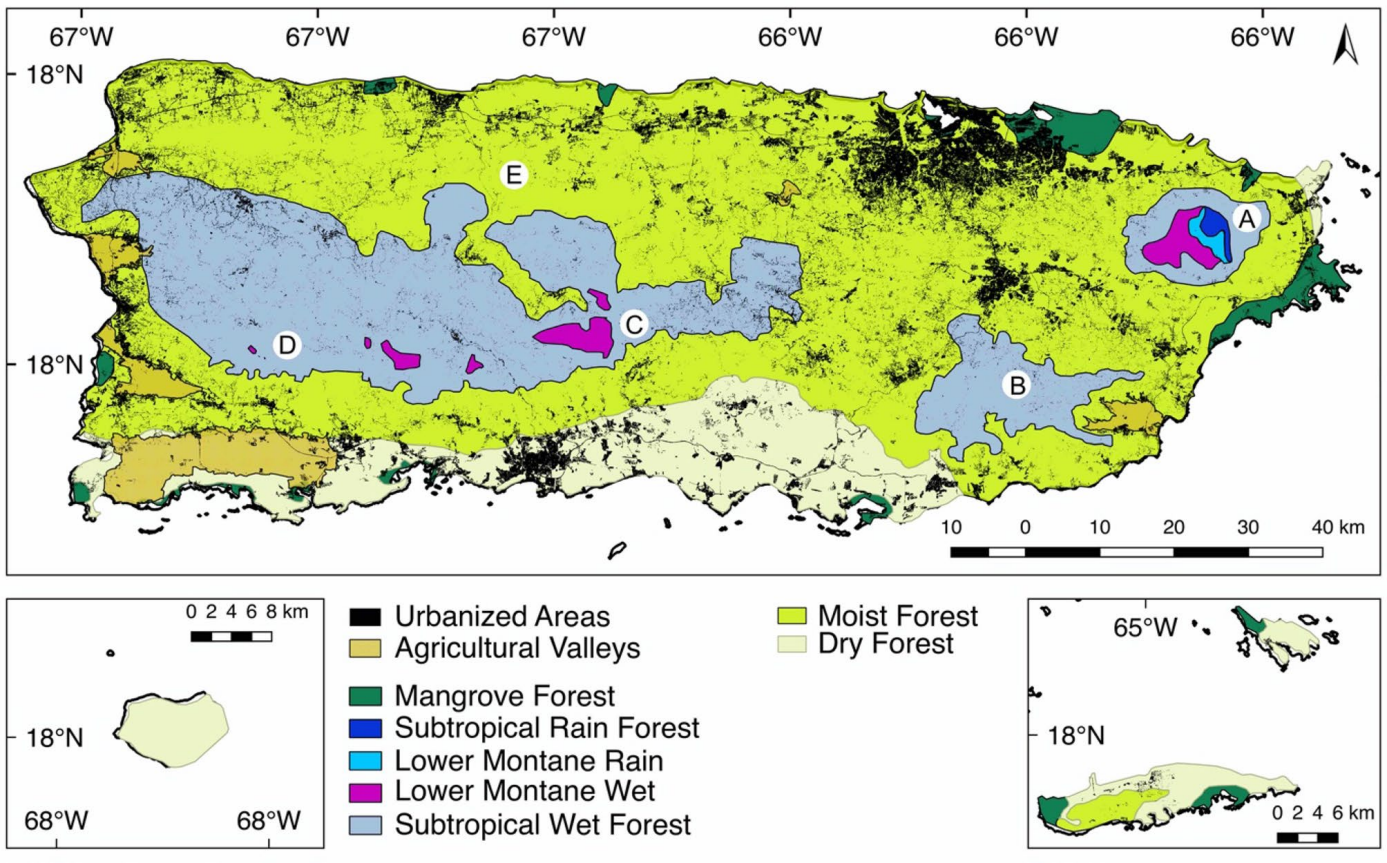
Forest types, agricultural valleys and urbanized areas of Puerto Rico^16,18^. (**A**) El Yunque National Park protects 5 different kinds of forests and many smaller ecosystems; (**B**) Carite State Forest; (**C**) Toro Negro State Forest; (**D**) Maricao State Forest and adjacent areas; (**E**) Río Abajo State Forest and adjacent areas. This map was created using QGIS v2.18.28^23^ (http://qgis.org). Land use data and categories use the Puerto Rico GAP Analysis Project datasets^19,20,24,25^ (https://data.fs.usda.gov/geodata/other_fs/IITF/index.php).

Combined, the protected and rustic areas form corridors that span from east to west and connect all forest types and protected areas in the main island of Puerto Rico. The east coast of Puerto Rico includes two important and constantly studied protected areas. El Yunque National Forest, itself the largest protected area of the island, and the nearby Carite State Forest (Fig. 1A and B, respectively) contain subtropical wet and rain forests and lower montane wet and rain forests. Notwithstanding, these large protected areas are under anthropogenic pressure and are affected by urbanization^17^. These areas in the east could lose connectivity between themselves and other protected areas in the center and west of the island, specifically the Toro Negro State Forest (Fig. 1C) which connects to the Maricao State Forest (Fig. 1D) and the Río Abajo State Forest (Fig. 1E). These central and western localities encompass subtropical wet and lower montane wet and rain forests that are only connected to the eastern forests by rustic areas.

The local fauna of the island is affected by anthropogenic disturbance, but also endure the seasonal pass of hurricanes and tropical storms, which may lead to population declines and potentially an increased risk of extirpation. In Puerto Rico, hurricanes have caused the decline or the potential extirpation of some bat species^26–31^. These effects have been observed in the most abundant and generalist species, *Artibeus jamaicensis*, and in the endemic and far less abundant, *Stenoderma rufum*, whose populations took nearly 5 years to recover to pre-hurricane levels following hurricane Hugo in 1989^26,27^. In Puerto Rico, this species primarily roosts in trees and occurs in forests throughout the island^15,32^. Drastic declines of this species in Puerto Rico could potentially affect populations in the smaller Virgin Islands, where it is considered rare^15,33^, and further reducing the likelihood of post-hurricane recovery via recolonization from this source population. The negative effects of hurricanes on *S. rufum* in Puerto Rico could also be exacerbated by the poor continuity of forest patches connecting local demes between the east and west of the island.

We aimed to model the population connectivity of *Stenoderma rufum* on Puerto Rico to examine the potential effects of anthropogenic (i.e., forest fragmentation) and natural (i.e. hurricanes) pressures. Developed and urbanized, or protected areas provide a proxy for habitat fragmentation, thus we measured anthropogenic effect using the resistance (or permeability) to animal movement provided by different land use categories. Given the roosting ecology, low population density, and slow post-hurricane recovery of *S. rufum*, we hypothesized that this species is adapted to dense forests that can be disrupted by anthropogenic or natural disturbance. Herein, we provide (1) an inference of the potential areas that offer the highest connectivity, (2) documentation of possible corridors with suitable habitats that connect protected areas, and (3) examination of the potential effect of hurricanes on the connectivity of *S. rufum* across Puerto Rico. Our population connectivity modeling approach sheds light into the importance of different forested areas for future conservation efforts of this insular endemic species and potentially other vertebrates.

## Results

**Figure 2:**
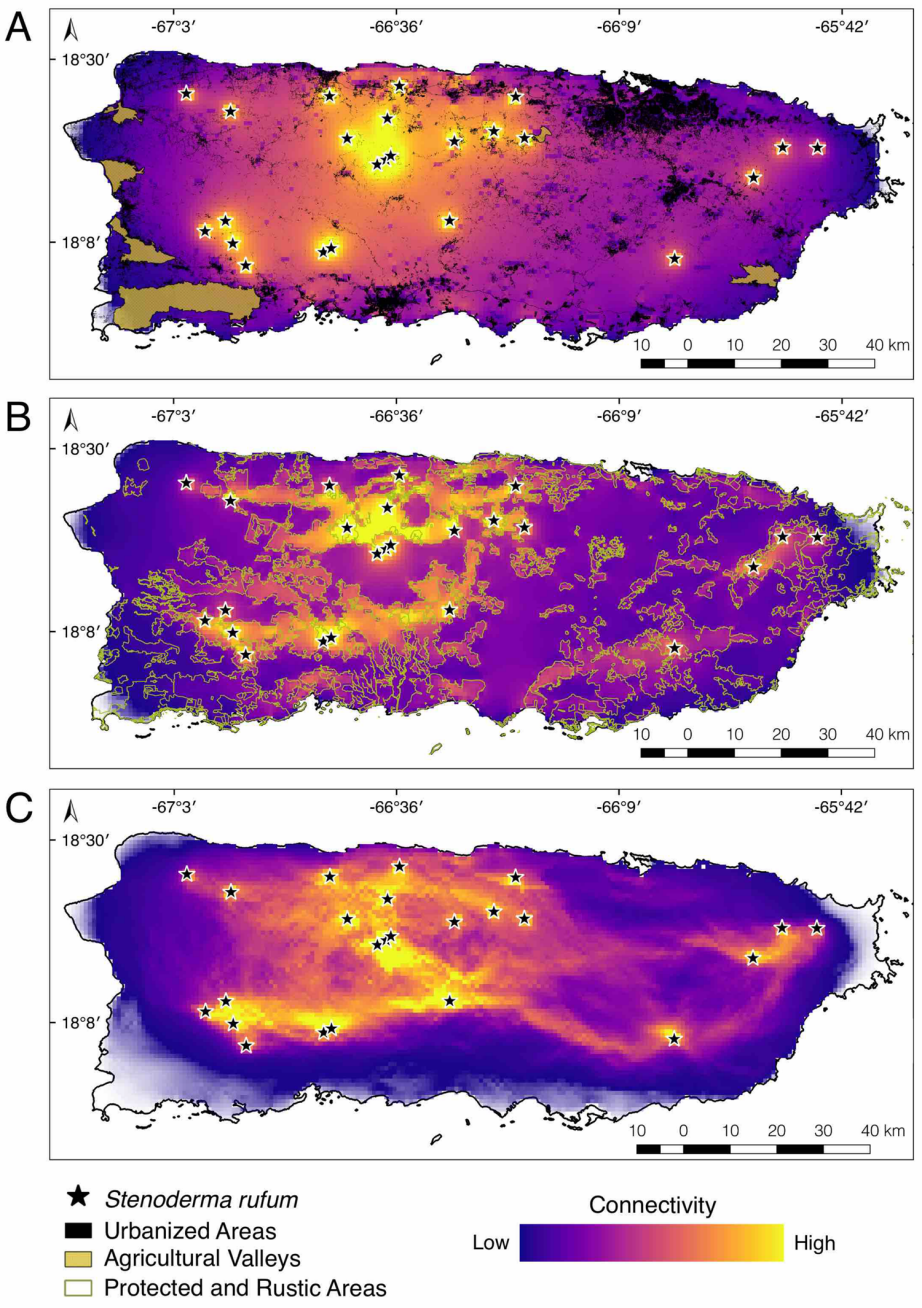
Landscape connectivity of *Stenoderma rufum* across the island of Puerto Rico estimated in Circuitscape. Three models were developed using (**A**) resistance from land use data, (**B**) conductance mediated by protected areas, and (**C**) habitat suitability derived from an ecological niche model. Army green outlines in panel B represent protected areas. Warmer colors indicate higher levels of connectivity, especially located within protected areas; white areas denote no connectivity. This map was created using QGIS v2.18.28^23^ (http://qgis.org). Land use data and categories use the Puerto Rico GAP Analysis Project datasets^19,20,24,25^ (https://data.fs.usda.gov/geodata/other_fs/IITF/index.php).

The different connectivity analyses of *Stenoderma rufum* showed four important areas that promote connectivity among roosting localities across the island (Fig. 2; refer to Fig. 1 for name places). The three analyses suggest limited connectivity between the eastern localities of El Yunque National Forest and Carite State Forest. Furthermore, both of these eastern localities show low connectivity with the central and western localities, i.e., Río Abajo and Toro Negro State Forests and Maricao State Forest, respectively.

When land use alone was included as a resistance layer, i.e., differential permeability in the landscape depending on the type of land use, the model produced an even connectivity gradient across the island (Fig. 2A). This gradient generally showed lower connectivity in the coastal areas that gradually increased towards inland forested areas. As predicted, only the urbanized, developed, and agricultural areas showed gaps in connectivity among *S. rufum* localities. This depicted a more fragmented landscape in coastal and eastern areas, while an increased in connectivity was observed among localities that cluster closer together in forests. Connectivity was concentrated primarily in the west of Puerto Rico, where urbanization and habitat fragmentation are lower than in the eastern areas^17^ (Fig. 2A).

We observed a similar pattern to the one described above when using protected and rustic areas as conductance surfaces to model the connectivity of *S. rufum*. Localities within protected forests in the west of Puerto Rico were well connected from north to south but disconnected from the scattered localities in the east (Fig. 2B). Despite the expansiveness of protected and rustic areas in the east including El Yunque National Forest and Carite State Forest, these lacked intermediate suitable forests that can act as ecological corridors to connect with the central and western regions of the island.

The ecological niche model (ENM) of *S. rufum* revealed that suitable habitat for this species concentrates in subtropical moist forest, particularly throughout lower montane, rain and moist forests (Supplementary Fig. S1). Habitat suitability decreased rapidly over coastal areas, lowlands, agricultural valleys, and subtropical dry forests. The variables that contributed most to the model were the mean temperature of the wettest and warmest quarters, precipitation of the driest quarter and annual precipitation. This model showed an overall good performance (AUC = 0.821; B_cont_ = 0.717). When the habitat suitability estimated from the ENM was used as a conductance layer, two main corridors were observed via the north and south of the island that link El Yunque National Forest with the western forests stretching to Toro Negro, Río Abajo, and Maricao State Forests (Fig. 2C). We found more north to south connections between the State Forests to the western part of the island, whereas El Yunque National Forest connected to the nearby Carite State Forest only by a narrow strip of suitable habitat running parallel to the southeastern coast (Fig. 2C). The western forests (i.e., Maricao, Río Abajo, and Toro Negro) connected north to south creating a loop of forests with multiple narrow corridors (Fig. 2C). This loop overlaps with the connectivity corridor created by protected and rustic areas (Fig. 2B).

**Figure 3:**
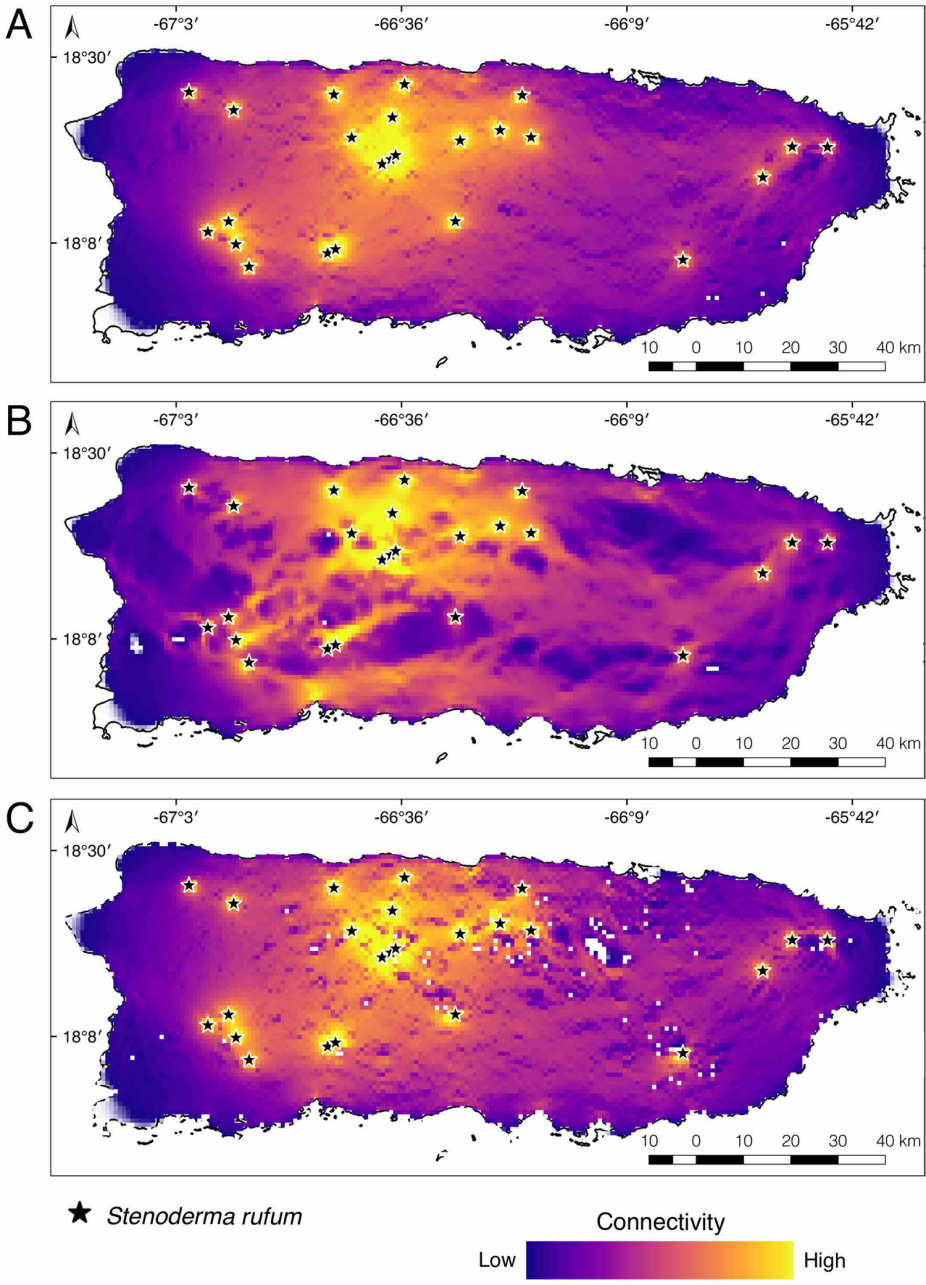
Connectivity of *Stenoderma rufum* based on conductance calculated using the Normalized Difference Vegetation Index (NDVI) values from (**A**) January 2017, (**B**) October 2017, and (**C**) January 2020 to reflect availability of vegetation cover before and after hurricanes Irma and Maria. This map was created using QGIS v2.18.28^23^ (http://qgis.org).

Connectivity analyses also reflected the potential negative effects of hurricanes Irma and Maria on *S. rufum* across the landscape (Fig. 3). Estimates of connectivity using pre-hurricane NDVI data (i.e., January 2017) as a conductance grid showed an even connectivity gradient throughout the island (Fig. 3A). This pattern was congruent with our expectations based on models produced using land use data. There was a decrease in vegetation cover immediately after the hurricanes^34^ (i.e., October 2017). This loss of vegetation cover disrupted connectivity and created gaps from north to south among Toro Negro, Río Abajo, and Maricao forests in the west (Fig. 3B). Models produced using NDVI data 14 months post-hurricane (i.e., January 2020) showed that forest connectivity increased nearly to pre-hurricane levels. We estimated that on average, the vegetation cover around localities where *S. rufum* has decreased by 35% after October 2017, with some localities inside El Yunque National Forest, Toro Negro, Carite and Maricao State Forests showing a decline in vegetation cover ranging from 70 to 90%.

## Discussion

The current biodiversity crisis stems from several causes, among them, habitat fragmentation is one of the main drivers of biodiversity loss in continental and insular mammal fauna^1^. Habitat fragment size and isolation within the landscape are important factors for quantifying the response of bat populations to habitat loss. Urbanization and loss of vegetation cover either via anthropogenic or natural causes may affect species in different ways^2^. For example, while specialist and/or rare species could be displaced, common species may flourish or recover easily, effectively modifying the composition of insular bat communities. Although the current population trend of *S. rufum* is unknown^35^, our results suggest that land use, natural disasters, and the availability of suitable habitat remaining play a role in maintaining the connectivity across the landscape for this bat. The maintenance of connectivity in *S. rufum* is important because of its solitary habits, ephemeral roosting preferences, and small home range of ca. 2 ha^26,36^. Until 30 years ago, *S. rufum* was known in Puerto Rico from only two localities. The rareness of this species and its potential to become extirpated from forests where it was once abundant make it imperative to better understand how *S. rufum* maintains its resilience in the face of habitat loss.

**Figure 4:**
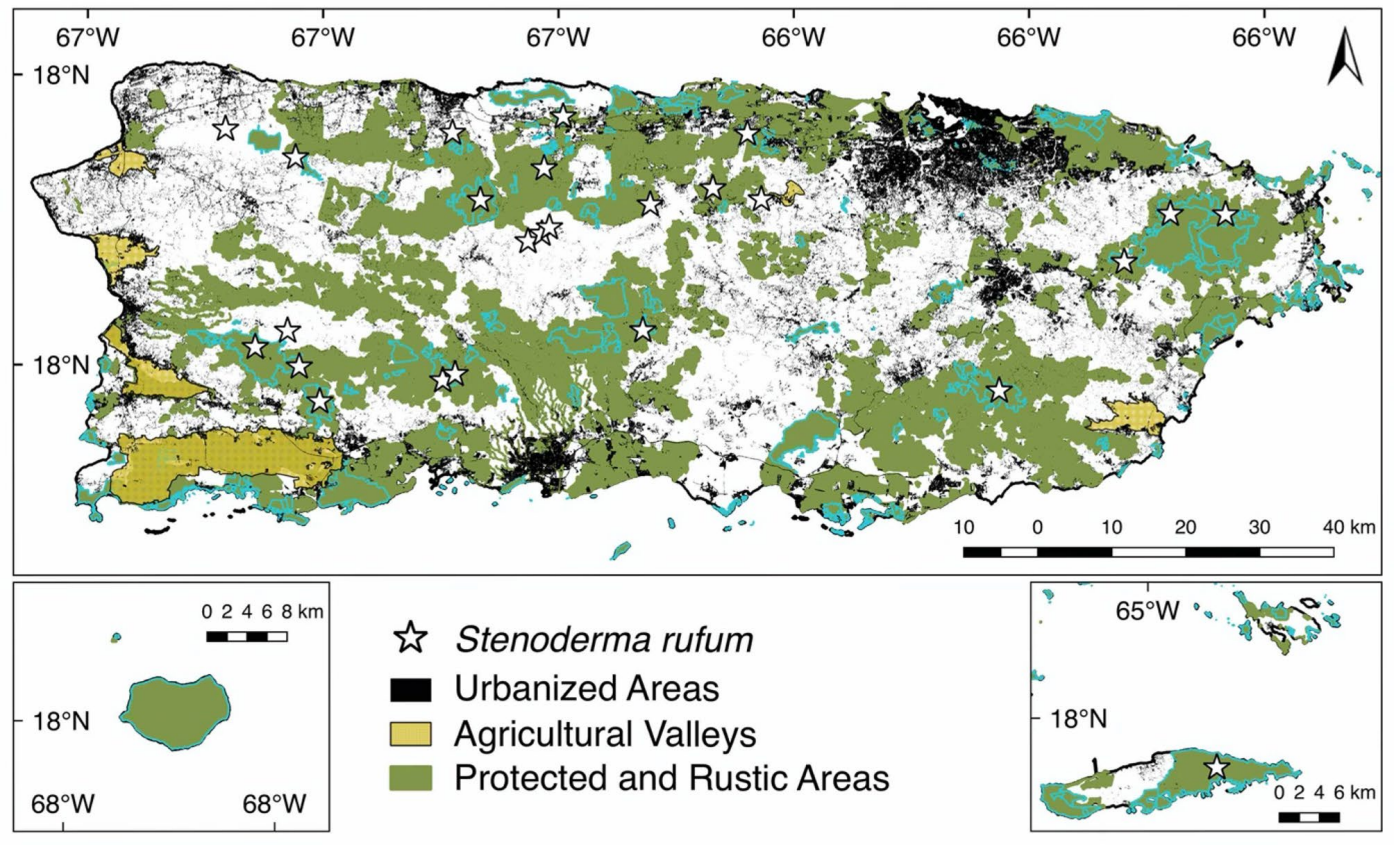
Record localities (i.e., stars) of *Stenoderma rufum* used to produce connectivity models in Puerto Rico. Map shows urbanized areas, agricultural valleys, and protected and specially protected rustic areas that were used for calculating functional connectivity. Light blue contours denote the protected areas following Gould (2009). This map was created using QGIS v2.18.28^23^ (http://qgis.org). Land use data and categories use the Puerto Rico GAP Analysis Project datasets^19,20,24,25^ (https://data.fs.usda.gov/geodata/other_fs/IITF/index.php).

The model that takes into account the suitable habitat available to *S. rufum* irrespective of land use (Fig. 2C) presents the most likely forest corridors for the species because it shows a hypothesis of connectivity based on abiotic requirements. Conversely, the models constructed using land use data and vegetation cover (Figs. 2A, 3A–C), should be taken as a best case scenario of connectivity because they assume that the species could move across any habitat in the landscape. Even though the latter two models represent gradients of connectivity throughout the island, they do not account for suitable habitat. Our results nevertheless highlight the importance of the western protected and specially protected rustic areas in Puerto Rico for providing connectivity corridors of suitable habitat between the north and south coasts. Furthermore, these findings emphasize the need for the preservation of the suitable habitat of *S. rufum* to ensure the connectivity among local demes. Under the different modeled scenarios, the protected areas in the west always remain connected, which may emphasize the importance of underdeveloped or low urbanized areas to maintain corridors for bat population connectivity (Figs. 2, 4). Notably, the effect of urbanized areas in restricting the connectivity of *S. rufum* is high, and models based only on habitat suitability show little to no connectivity around urban centers (Fig. 2A, C). The only decrease in connectivity in this region was observed when models included vegetation cover data immediately after a natural event (Fig. 3; i.e., September 2017) that removed from 70 to 90% of the vegetation cover around localities where *S. rufum* has been documented within protected areas. The influence of a high intensity category-4 storm, hurricane Maria, underscores the potentially detrimental effects that loss of vegetation cover can have on bat populations.

Several important points can be gleaned from the modeling scenarios we presented. First, habitat on the east of Puerto Rico could be highly susceptible to future changes in land use and urbanization because of the already fragmented nature of the landscape. Second, the interconnecting corridors between the preserved forests to the east and west can be vital for maintaining gene flow in *S. rufum* and may help in species recovery after natural disturbances. Third, the western forests (i.e., Maricao, Río Abajo, and Toro Negro) form a well-connected network of habitat and each may serve as a hub or refugium for the long-term persistence of populations. This is particularly important in the center of the island, where the corridor narrows (Fig. 2B). If these protected areas maintain ecological connectivity, they could represent important movement corridors for local fauna.

Recently, Guzmán-Colón et al.^17^ proposed a connectivity model using only protected areas and forest patches representing the home ranges of several vertebrates in Puerto Rico. Their findings are highly congruent with our results, despite being produced using different methods. Guzmán-Colón et al.^17^ found that the human footprint (habitat transformation) in forest patches outside of the protected areas in the central and montane zones is “very low”, but the scattered pattern of these patches is indicative of habitat fragmentation and low connectivity. Coastal areas had a “very high” human footprint scoring and 18% of the protected area had “medium” to “very high” values of habitat transformation. Furthermore, they documented a network hub in the central northwest of Puerto Rico. Although the analysis of Guzmán-Colón et al.^17^ focused on other vertebrates that are potentially less vagile than bats, their results support our conclusions that the western forests provide a local hub for *S. rufum* connecting with other areas of the island and that the eastern localities could be under the pressure of habitat fragmentation and loss of connectivity.

In contrast to Guzmán-Colón et al.^17^, we combined data from rustic areas in addition to protected forests to provide estimates of connectivity. Because of their designation, rustic areas are under less pressure of development in the future. Despite that these rustic areas have a relatively low human footprint, they could still be used for agricultural purposes in the short term. We believe that rustic areas are of high importance for vertebrate populations because they encompass a significant amount of suitable habitat that can improve connectivity among local demes across Puerto Rico. These rustic areas, therefore, represent important points for land conservation efforts.

Studies documenting bat responses to habitat fragmentation often show species specific results. On landbridge islands of Lake Gatún, Panama, researchers have documented that abundances of insectivorous Neotropical bats often decline in response to habitat fragmentation, whereas plant visiting bats tend to increase^37,38^. In both cases, previous results suggested that small forest fragments seem to hold significant conservation value and forest integrity was proposed to be of high conservation priority. On Puerto Rico, and other Caribbean islands, forest integrity is disrupted by hurricanes, which pose intense and frequent natural disturbances in addition to ongoing anthropogenic forest fragmentation. These hurricanes cause rapid changes that alter forest integrity and reduce vegetation cover particularly affecting old forest stands more significantly than younger ones^39^. The newly recovered forest is structurally different than before, and some of the areas most affected by hurricane María in 2017, and having the longest lasting residual effects, include El Yunque National Forest, and Carite, Maricao, Río Abajo, and Toro Negro State forests (see Fig. 4 in Feng et al.^39^).

Previously, the bats of Puerto Rico have shown a decline in response to hurricane activity. Bat populations showed a significant reduction in abundance and sampling across habitats revealed a decrease in species richness^29^. In contrast to the patterns described above based on habitat fragmentation on landbridge islands of Panama, the effects of hurricane Georges in 1998 produced the opposite short-term effect on Puerto Rico, with insectivores increasing in abundance and plant visiting bats showing a decrease. In addition to shifts in species composition, recent studies highlight the susceptibility of bats to rapid disturbance with the possible extirpation of the frugivorous *Artibeus jamaicensis* following hurricane Maria^31^. Gannon and Willig^26^ showed that population recovery of *S. rufum* took 5 years to reach pre-hurricane levels at El Yunque National Forest after hurricane Hugo in 1989. Following hurricane María, *S. rufum* was not present on forest fragment sites surveyed near the eastern metropolitan areas where it had been captured before (Rodríguez-Durán unpub. data). Given the magnitude of forest cover loss and the post-hurricane disruption of habitat corridors (Figs. 2, 3), it is likely that the absence and slow recovery of *S. rufum* from eastern Puerto Rico results from the lack of connectivity across the landscape. Maintaining the habitat corridors coupling western forests with the more isolated eastern forests in Puerto Rico could help facilitate the recolonization of *S. rufum* and may be key to maintaining resilience among populations.

Our study takes a first step into understanding the potential effects of land use, habitat suitability, and natural disasters to estimate intra island population connectivity in bats, the only native mammals remaining on many Caribbean islands^40^. One notable finding of our models is that suitable interconnected habitat for *S. rufum* is located primarily on the west of Puerto Rico, despite that information about the ecology of this bat has primarily focused on the El Yunque National Forest population^15^. It is important to note that our models estimate connectivity only from the perspective of habitat availability and additional data could help clarify the use and importance of habitat corridors on the island. A clear next step for validating our findings is to incorporate landscape genetics to untangle whether the corridors estimated herein help maintain gene flow of *S. rufum* across the island. Deciphering whether populations in each of the main protected forests hold important genetic variability and the directionality of gene flow can shed light into the source-sink dynamics of local demes following habitat disruption by hurricanes. Integrating this approach with connectivity models can help identify areas where populations might shift their ranges as suitable habitat changes under different climate change scenarios^41^. Additional studies on *S. rufum* should include a boots-on-the-ground approach targeting the habitat corridors we predicted herein and tracking the movement of individuals across these corridors to confirm their suitability as communing or foraging areas.

Bats provide important ecosystem services in their role as pollinators, seed dispersers, predators of arthropods and in the flux of energy over long distances in the ecosystem^42^. However, anthropogenic factors are evolving into ever increasing challenges for the conservation of these mammals. A recent evaluation of factors affecting bats throughout the Neotropics point to habitat loss and fragmentation, and roost destruction as major threats to bat conservation^43^. The models presented here offer a new approach for studying bat communities and highlight the potential value of rustic areas for increasing forest integrity and areas of significant conservation value for bats in Puerto Rico, and potentially other vertebrates elsewhere. These models could be useful for decision-makers to develop strategies that would facilitate the conservation of critical habitats.

## Methods

We modeled the landscape connectivity of the red fig eating bat (*Stenoderma rufum*), an endemic species on Puerto Rico, based on occurrence localities across the island. Our area of interest spans the main island of Puerto Rico, covering all seven types of forest and areas under any form of protection. Focusing on *S. rufum* in Puerto Rico was advantageous because this island has the largest, best monitored, and most continuous populations of the species compared to satellite populations in the Virgin Islands^27,28,31,33,44–47^. Additionally, land use categorization and forest type distribution in Puerto Rico are well known and the Puerto Rico Gap Analysis Project has available datasets ^18,19^. Our landscape modeling approach used electrical circuit theory implemented in the software Circuitscape v4.05 to examine the landscape connectivity of *S. rufum* under different scenarios^48,49^. Circuit theory calculates the flow of current between pairs of nodes (i.e. species localities) in the landscape, the latter being a continuous surface with resistance or conductance values that indicate different potential pathways (i.e. habitat corridors) for animals to move through^49^. Resistance layers represent the opposition of the landscape to movement, while conductance layers are the reciprocal of the resistance and are analogous to habitat permeability. By calculating the flow of current across each node one can identify areas of interest that can potentially help maintain the population connectivity of a species across the landscape and prioritize areas for conservation^50–54^.

### Study area and data sources

The main island of Puerto Rico has an area of 8948 km^2^ of which 46% is dedicated to urban and barren areas, and agricultural valleys and pastures; forests, woodlands and shrublands account for 54% of the total area^19^. To the east, the island is under pressure by rapid urban development and an increasing human footprint^17^. Although the protected and rustic areas offer connectivity between the main forests through the island (Fig. 1).

We obtained all known georeferenced localities of *Stenoderma rufum* (N = 46) in Puerto Rico, including places of capture and roosts from the published literature and GBIF records^15,46,55^. We selected 26 of these 46 localities as nodes for our connectivity analysis because they represented roosts and localities of consistent captures^15^. The complete dataset of 46 localities also included incidental and one-time reports. This approach was critical to our study, because it provided a measure of the environmental niche that takes into account bat movement through the island (represented in the form of incidental records and captures in bat surveys) while also incorporating the connectivity of known long term capture areas. Bat surveys and sampling across Puerto Rico is even, which reduced sample bias. The spatial data was filtered excluding localities that were less than 1 km^2^ apart to account for issues of spatial autocorrelation. This filtering scheme was appropriate because of the small home range of *S. rufum* (ca. 2 ha^23,34^). For the connectivity analyses, we used land use and protected areas data from the Puerto Rico Gap Analysis^18,20,24,25^. Land use grids represented built area and are available at a cell size of 300 × 300 m. Data for protected areas was extracted from different pre-classified polygons^20^ (see Models of Population Connectivity). All data were scaled to a resolution of 30 s using the raster calculator in QGIS v2.18.28 ^23^. Also, publicly available climate and elevation data grids from WorldClim v2^56^ were used as environmental variables to estimate the ecological niche of *S. rufum*. Finally, three Normalized Difference Vegetation Index (NDVI) layers representing vegetation cover information from January 2017, October 2017, and January 2020 were used to examine the effects of forest cover change on connectivity^57^.

### Models of population connectivity

We modeled the connectivity among 26unique localities of *S. rufum* (Fig. 2, Supplementary Table 1) under 4 scenarios, (1) connectivity based on a resistance layer using different categories of land use; (2) connectivity using the protected areas as a conductance layer; (3) connectivity of *S. rufum* based on a conductance layer that accounted for habitat suitability estimated using ecological niche modeling (ENM); and (4) connectivity based on a layer of conductance using NDVI across three time snapshots that reflected forest cover change following a recent natural disturbance (i.e., hurricanes Irma and Maria) and its effect on the landscape connectivity. All connectivity models followed the steps: (1) input data selected as raster, (2) the chosen modelling approach was pairwise across all localities. Additional settings included average resistance for connection between cells in scenario one and conductance for connections between cells in scenarios two to four. We connected all raster cells to eight neighbors instead of four, and chose the voltage, cumulative, and maximum current maps as outputs.

#### Effects of land use on bat connectivity

To understand the effect of land use on bat connectivity, we used the land use classification grid of the Puerto Rico Gap Analysis Project^19,24,25^ to extract the geographic data of urban centers, agricultural valleys, and protected areas (Fig. 4). To reflect the increasing levels of resistance represented by habitat modification (i.e. agriculture and urbanization), we followed the human footprint and habitat modification of Guzmán-Colón et al.^17^ and equate a high human footprint and modification, and habitat fragmentation value with a higher resistance (opposition to animal movement). Because of their low human footprint, the conductance layer of protected and rustic areas was considered more permeable to facilitate animal movement.

We reclassified cell values from low to high resistance using a value of 1 for any forest, rural and non-urbanized or agricultural area. This provided the least resistance to bat movement among habitats. We also set a resistance of 3 for agricultural valleys because they can maintain some permeability to movement despite the lack suitable foraging habitat for bats, being three times more opposing to movement for a forest specialist bat. Finally, we used a resistance of 5 for highly urbanized areas defined as any built and non-vegetated areas resulting from human activity^25^. These typically correspond to large urban areas that represent the highest resistance to movement that *S. rufum* would encounter given the documented susceptibility of this species to large scale habitat disturbance. In the models, resistance values represent classified habitat with the lowest permeability to bat flight, that is, they do not fully restrict movement even if set at the highest resistance levels for urbanized areas. This approach allowed us to understand how resistant the landscape is to bat mobility. We calculated the connectivity also in terms of the landscape being a conductive surface in which protected and rustic areas provide higher conductance and therefore are more permeable than developed areas. Here, grid values were classified as 0.5 for urban areas and agricultural valleys (i.e., low conductance), a value of 2 for rustic areas because they share a direct edge with developed and urbanized areas (i.e., intermediate conductance), and a value of 3 for protected areas (i.e., high conductance).

#### Land connectivity accounting for habitat suitability

To examine the connectivity based on the suitable habitat required by *S. rufum*, we first estimated habitat suitability using an Ecological Niche Modeling (ENM) approach under a maximum entropy framework^58–62^ using data from GBIF and published literature (n = 46)^15,55^. Specifically, we modeled the potential distribution of *S. rufum* to use as input for a conductance model. The ENM was generated using MaxEnt v3.4.1^63,64^ and included 19 current climate variables and elevation available in WorldClim v2^56^. We chose the best parameter settings for MaxEnt modeling using the R^65^ package ENMeval^66^. Five different feature class combinations: L, LQ, H, LQH, and LQHP (L = linear, Q = quadratic, H = hinge, P = product) and regularization multipliers from 0.5 to 2 (with a step value of 0.5) were examined. The selected best fit model parameters included Linear features and a regularization value of 0.5. These parameters were used to produce a final model with observations randomly partitioned into 75% training and 25% testing sets ran over 100 bootstrap replicates for model validation. Model performance was evaluated using the AUC statistic estimated in MaxEnt. Independently, we also evaluated model performance using the continuous Boyce index (B_cont_)^67^, a metric that is suitable for presence only datasets. Values of the Boyce index range from − 1 to 1, where negative scores represent a model with counter predictions of poor-quality habitat where presences are frequent, values closer to 0 represent model performance no different than random, and positive values represent a model consistent with the distribution of presences in the dataset. Thus, Boyce index values closer to 1 are indicative of good model performance. The resulting raw output average habitat suitability map produced from the ENM of *S. rufum* was used as a conductance grid*.* We calculated conductance based on this suitable habitat connectivity between all pairs of 26 unique localities of *S. rufum.*

#### Effects of natural disturbance on bat connectivity

We used NDVI data from three specific time periods, January 2017, October 2017 and January 2020^57^ to create three independent conductance models. These three models were used to compare the connectivity among localities of *S. rufum* before, immediately after, and 26 months after the most recent large scale storms that affected Puerto Rico in September 2017 (i.e., hurricanes Irma and Maria). We used the data range of NDVI values in our grid as conductance values for Circuitscape, with higher NDVI values representing better conductance across the landscape.

## Supplementary Information

Supplementary Information.

## Data Availability

All geographic occurrence data is freely available through GBIF.org^49^ and the published literature. We make available these geographic coordinates in the Supplementary Material.
