# Supplementary material for: Effect of land use, habitat suitability, and hurricanes on the population connectivity of an endemic insular bat
Source: Sci Rep. 2021 Apr 27;11:9115. doi: 10.1038/s41598-021-88616-7 (PMC8079362; doi:10.1038/s41598-021-88616-7)
Supplement: Supplementary file 1 — Supplementary Information. [file 41598_2021_88616_MOESM1_ESM.pdf]

Effect of land use, habitat suitability, and hurricanes on the population connectivity of an endemic insular bat

Camilo A. Calderón-Acevedo, Armando Rodríguez-Durán and J. Angel Soto-Centeno

Supplementary information

Supplementary Figures

Supplementary Figure S1. Ecological niche model of *Stenoderma rufum* in Puerto Rico based on 1 elevation layer and 19 WorldClim climate variables[1] summarizing regional temperature and precipitation. This map created using QGIS v2.18.28[2] (http://qgis.org).

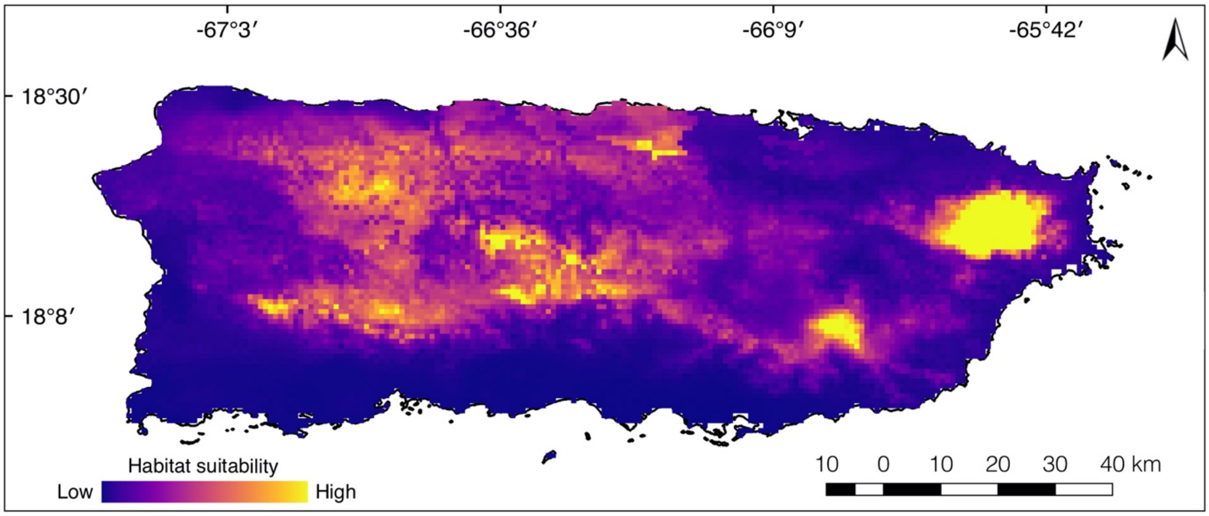

Supplementary Tables

Supplementary Table 1. Georeferenced localities of *Stenoderma rufum* used in the Environmental Niche Modeling analysis from Gannon et al. 2005[3] and Rodríguez-Durán & Feliciano-Robles 2016[4].

Localities	Latitude	Longitude	Source
Adjuntas	18.10968	-66.74767	Gannon et al. 2005
Adjuntas	18.117622	-66.731821	Gannon et al. 2005
Arecibo	18.42539	-66.73511	Gannon et al. 2005
Arecibo	18.33967	-66.69962	Gannon et al. 2005
Arecibo	18.38004	-66.61833	Gannon et al. 2005
Barceloneta	18.44606	-66.59406	Gannon et al. 2005
Canovanas	18.287248	-65.874342	Gannon et al. 2005
Canovanas	18.260475	-65.878782	Gannon et al. 2005
Corozal	18.340454	-66.341434	Gannon et al. 2005
Isabela	18.43061	-67.0245	Gannon et al. 2005
Isabela	18.39415	-66.93522	Gannon et al. 2005
Luquillo	18.320983	-65.749589	Gannon et al. 2005
Maricao	18.17301	-66.94546	Gannon et al. 2005
Maricao	18.152183	-66.986714	Gannon et al. 2005
Morovis	18.35457	-66.40319	Gannon et al. 2005
Orocovis	18.173414	-66.492497	Gannon et al. 2005
Patillas	18.096432	-66.03818	Gannon et al. 2005
Rio Grande	18.3216667	-65.82	Gannon et al. 2005
Sabana Grande	18.12733	-66.9306	Gannon et al. 2005
Utuado	18.28702	-66.63892	Gannon et al. 2005
Utuado	18.29651	-66.62119	Gannon et al. 2005
Utuado	18.30475	-66.6117	Gannon et al. 2005
Vega Baja	18.42417	-66.359216	Gannon et al. 2005
Vieques	18.140731	-65.367523	Gannon et al. 2005
Yauco	18.082935	-66.904471	Gannon et al. 2005
Ciales	18.333919	-66.483044	Rodríguez-Durán & Feliciano-Robles 2016

References

1. Fick, S. E. & Hijmans, R. J. WorldClim 2: new 1-km spatial resolution climate surfaces for global land areas. *Int. J. Climatol.* **37**, 4302–4315 (2017).
2. QGIS.org. QGIS Geographic Information System. (2016).
3. Gannon, M. R., Kurta, A., Rodríguez-Durán, A. & Willig, M. R. *Bats of Puerto Rico: an island focus and a Caribbean perspective*. (Texas Tech University Press, 2005).
4. Rodríguez-Durán, A. & Feliciano-Robles, W. Conservation Value of Remnant Habitat for Neotropical Bats on Islands. *Caribb. Nat.* **35,** 1–10 (2016).
